# Applications of Chitosan and its Derivatives in Skin and Soft Tissue Diseases

**DOI:** 10.3389/fbioe.2022.894667

**Published:** 2022-05-02

**Authors:** Yidan Xia, Dongxu Wang, Da Liu, Jiayang Su, Ye Jin, Duo Wang, Beibei Han, Ziping Jiang, Bin Liu

**Affiliations:** ^1^ Department of Hand and Foot Surgery, The First Hospital of Jilin University, Changchun, China; ^2^ Laboratory Animal Center, College of Animal Science, Jilin University, Changchun, China; ^3^ Department of Pharmacy, Changchun University of Chinese Medicine, Changchun, China

**Keywords:** chitosan, soft tissue disease, biological property, drug-delivery carrier, regenerative medicine

## Abstract

Chitosan and its derivatives are bioactive molecules that have recently been used in various fields, especially in the medical field. The antibacterial, antitumor, and immunomodulatory properties of chitosan have been extensively studied. Chitosan can be used as a drug-delivery carrier in the form of hydrogels, sponges, microspheres, nanoparticles, and thin films to treat diseases, especially those of the skin and soft tissue such as injuries and lesions of the skin, muscles, blood vessels, and nerves. Chitosan can prevent and also treat soft tissue diseases by exerting diverse biological effects such as antibacterial, antitumor, antioxidant, and tissue regeneration effects. Owing to its antitumor properties, chitosan can be used as a targeted therapy to treat soft tissue tumors. Moreover, owing to its antibacterial and antioxidant properties, chitosan can be used in the prevention and treatment of soft tissue infections. Chitosan can stop the bleeding of open wounds by promoting platelet agglutination. It can also promote the regeneration of soft tissues such as the skin, muscles, and nerves. Drug-delivery carriers containing chitosan can be used as wound dressings to promote wound healing. This review summarizes the structure and biological characteristics of chitosan and its derivatives. The recent breakthroughs and future trends of chitosan and its derivatives in therapeutic effects and drug delivery functions including anti-infection, promotion of wound healing, tissue regeneration and anticancer on soft tissue diseases are elaborated.

## 1 Introduction

Chitosan is a naturally occurring, newly identified cationic polysaccharide, which is a deacetylation product derived from chitin (Wang W. et al., 2020). Chitosan has been widely used in the medical field as a wound dressing because of its appreciable antibacterial activity (Matica et al., 2019). However, chitosan is poorly soluble and unstable in water; thus several chitosan derivatives have been developed (Shahid Ul and Butola, 2019). These derivatives were obtained by chemical modifications, which retained the effective biological properties of the parent chitosan while improving its physical and chemical properties (Ardean et al., 2021). Chitosan and its derivatives have been processed into hydrogels, sponges, microspheres, nanoparticles, and thin films for use as medical materials. These are widely used to treat different diseases, especially those of the skin and soft tissues, owing to the diverse properties of these compounds (Ma et al., 2017; Zhang N. et al., 2020; El Kadib, 2020; Hou et al., 2020; He et al., 2021).

Skin and soft tissue diseases include trauma, infections, and tumors of the skin, subcutaneous tissue, and fascia (Endo et al., 2019; Peetermans et al., 2020). Trauma to the skin, muscles, blood vessels, and nerves can be treated with chitosan and its derivatives as they promote wound healing (Guo et al., 2019; Alven and Aderibigbe, 2020; Rao F. et al., 2020; Zhao et al., 2021). Given that soft tissue infections such as those of the skin and subcutaneous tissues are caused by bacteria or fungi, chitosan and its derivatives can be used as dressings to treat infected wounds (Matica et al., 2019; Watkins and David, 2021). Soft tissue sarcomas are the most common malignancies of fat tissue, fascia, muscles, lymph nodes, and blood vessels, which always lead to a poor prognosis due to their insidious onset and rapid metastasis to distant organs. Chitosan and its derivatives exert antitumor activities and can, therefore, be potentially used in drug-delivery systems for the treatment of sarcoma (Maleki Dana et al., 2021). Besides, chitosan-based nanoparticles, sponges, films, hydrogels, and scaffolds have been used for soft tissue injury treatment (Oryan and Sahvieh, 2017; Hemmingsen et al., 2021; Rashki et al., 2021; Sun et al., 2021). Although chitosan and its derivatives have broad application prospects in the skin and soft tissue diseases, there is still a lack of review on this aspect. This review summarizes the sources, structures, biological characteristics, and different forms of drug carriers of chitosan and its derivatives. It also discusses the recent breakthroughs in the application of chitosan and its derivatives in preventing and treating trauma, infection, and tumor of skin and soft tissues.

## 2 Preparation of Chitosan and its Derivatives

Chitin is mainly obtained from the corneum of crustaceans, such as shrimp and crab shells, which are purified by chemical and biological extraction to remove protein and precipitate calcium carbonate (Younes and Rinaudo, 2015). Chemically, chitosan consists of 2-amino-2-deoxy-D-glycopyranose units linked by β (1→4) glycosidic bonds and is obtained by the chemical and enzymatic deacetylation of chitin (Supplementary Figure S1) (Santos et al., 2020). The unique structure of chitosan makes it insoluble in water and most organic solvents, limiting its scope of applications (Muxika et al., 2017). Chitosan has been chemically and biologically modified by acylation, carboxylation, alkylation, and quaternization to improve its solubility and prepare derivatives for comprehensive applications.

The biocompatibility and anticoagulation effects of N-acylated chitosan have been significantly improved over the years and can be used as a sustained-release drug in a clinical setting (Wang W. et al., 2020). A previous study confirmed that the antibacterial activity of water-soluble N-alkylated disaccharide chitosan derivatives against *Escherichia coli* and *Staphylococcus aureus* was significantly higher than natural chitosan at pH 7.0 (Yang et al., 2005). Carboxymethyl chitosan can affect its solubility in water across different pH by affecting the degree of carboxymethylation, thus prolonging the reaction time of the drug-delivery system (Shariatinia, 2018). Therefore, modifying chitosan through quaternization could significantly improve its water solubility, antibacterial effects, mucosal adhesion, and permeability, which are beneficial for designing medical dressings and drug carriers (Freitas et al., 2020). Chitosan and its derivatives exert antibacterial, antioxidant, and anticancer effects *in vivo* as drug carriers, highlighting their potential application in clinical diseases.

## 3 Biological Characteristics of Chitosan and its Derivatives

### 3.1 Antibacterial Activity

**FIGURE 1:**
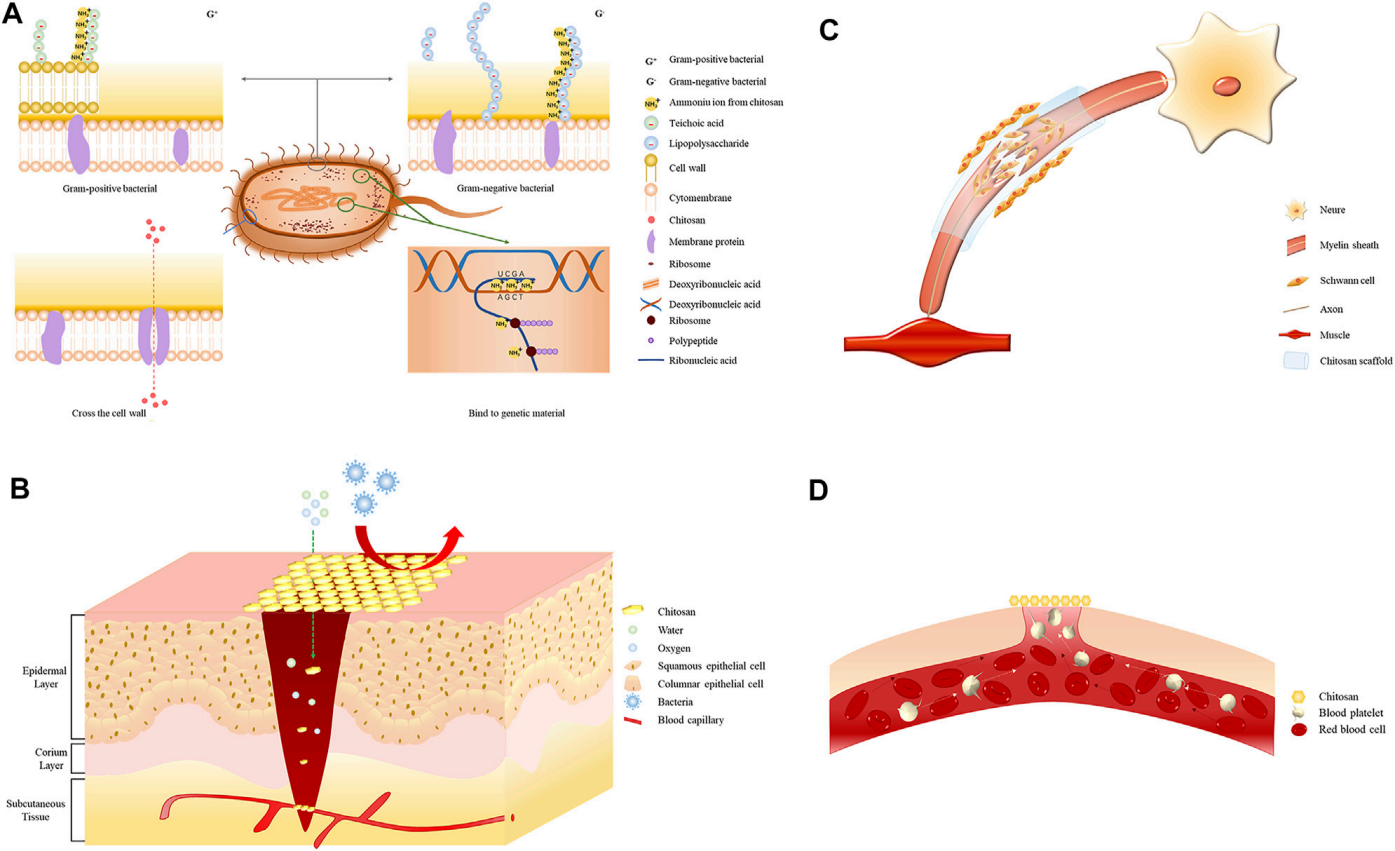
**(A)** Electrostatic interaction of the positively charged ammonium ion with the negatively charged teichoic acid in Gram-positive bacteria. The positively charged ammonium ion interacts electrostatically with the negatively charged phospholipid molecule in Gram-negative bacteria. Chitosan molecules enter through protein channels on the bacterial membrane and interfere with physiological functions. Electrostatic interaction of the positively charged ammonium ion with the negatively charged nucleic acid group. **(B)** Chitosan wound dressings allow the permeation of oxygen and water to keep the wound moist while preventing bacterial contamination and wound infection. **(C)** Chitosan promotes nerve regeneration by promoting Schwann cell proliferation. **(D)** Chitosan promotes erythrocyte aggregation and platelet adhesion.

The amino group in the chitosan structure can be converted to a positively charged ammonium ion, which confers cationic properties to chitosan (Fakhri et al., 2020). The cell walls of Gram-positive bacteria are mainly composed of teichoic acid, which is negatively charged and can react with chitosan via electrostatic interactions, leading to the destruction of the bacterial cell wall, loss of cellular function, and ultimately cell death (Abd El-Hack et al., 2020). The ammonium ions in chitosan interact with the anions of lipopolysaccharides present on the outer membrane of Gram-negative bacteria, leading to a bacteriostatic effect (Ardean et al., 2021). Additionally, chitosan can cross bacterial cell membranes and interfere with the transcription and translation of genetic material, thus affecting the normal cellular function (Figure 1A) (Verlee et al., 2017). The antibacterial performance of chitosan against *Staphylococcus epidermidis* significantly increased when the compound was functionalized with catechol, as demonstrated by a decrease in the minimum inhibitory concentration of the polymer (Amato et al., 2018). The antibacterial properties of chitosan when formulated as hydrogels, films, sponge wound dressings make it a good wound-treatment material for the prevention and treatment of infections. A novel lignin-chitosan-PVA composite hydrogel designed as a wound dressing shows good adsorption capacity and bacteriostatic effects (Zhang Y. et al., 2019). Chitosan films containing glycerin as a strengthening agent can be used as a wound dressing to inhibit bacterial infections (Ma et al., 2017). The composite sponge prepared using hydroxybutyl chitosan and chitosan combined the hydrophilic properties of hydroxybutyl chitosan and the antibacterial properties of chitosan, highlighting its potential as a wound dressing (Hu S. et al., 2018). The successful use of these preparations in treating skin and soft tissue infections is indicative of the antibacterial effects of chitosan.

### 3.2 Antioxidant Activity

The body maintains an oxidation balance under normal physiological conditions. When the antioxidant capacity is not adequate to combat the sudden increase in free radicals, the surplus free radicals lead to cell injury, metabolic disorders of the cellular macromolecules, and the occurrence of skin and soft tissue diseases (Sztretye et al., 2019). The antioxidant properties of chitosan are attributed to the amino and hydroxyl groups in its molecular chain, which can effectively scavenge excessive free radicals in the human body (Muthu et al., 2021). The antioxidant activity of chitosan mainly depends on its relative molecular weight and the level of acetylation (Abd El-Hack et al., 2020; Cabañas-Romero et al., 2020). Chitosan shows a greater ability in scavenging free radicals having relatively low molecular weights and higher levels of acetylation (Negm et al., 2020). Chitosan derivatives obtained by chemical modification can improve the antioxidant capacity of polymers and increase their application over a range of fields (Hao et al., 2021). Chitosan composite films prepared with ascorbate have stronger DPPH radical–scavenging ability and improved ability in resisting ultraviolet-visible light and visible light (Tan et al., 2020). Chitosan derivatives containing Schiff’s base and a quaternary ammonium salt exhibit stronger antioxidant capability than chitosan due to the presence of hydroxyl and halogen groups (Wei et al., 2019). Novel chitosan derivatives containing sulfur salts have DPPH-, hydroxyl-, and superoxide radical-scavenging capacities of higher than 90%, without any associated cytotoxicity (Sun et al., 2020). Chitosan nanoparticles synthesized by doxorubicin can significantly enhance the scavenging ability of free radicals and reduce the cell viability of liver, stomach, lung, and breast cancer cells, which can be used as a potential drug carrier for tumors (Mi et al., 2021). The antioxidant capacity of chitosan can be regulated by adjusting its molecular weight, acetylation level, and the extent of chemical modification, thereby conferring tremendous application prospects in medical cosmetology and the treatment of soft tissue diseases and tumors.

### 3.3 Anticancer Activity

Cancer is one of the most challenging conditions to cure, with surgical resection being the most efficient and effective management technique. The development of targeted drugs provides new ideas to treat cancer; however, several drugs have poor bioavailability, low selectivity, and poor stability in tumor tissues (Kandra and Kalangi, 2015). Chitosan derivatives incorporated into the nano drug-delivery systems have emerged as one of the most advanced delivery systems in the biomedical field. This technology is associated with minimum systemic toxicity and maximum cytotoxicity to the tumors and cancer cells and is the most promising targeted therapy in cancer (Verlee et al., 2017). Chitosan can directly inhibit the growth of tumor cells, induce cell necrosis and apoptosis, and enhance immunity to achieve its antitumor effect (Yu et al., 2022). The chitosan-based nanoparticles could selectively permeate cancer cells and precisely exert their effects by continuously releasing the loaded drugs while maintaining drug stability (Kamath and Sunil, 2017). N, O-carboxymethyl chitosan/multialdehyde Guar hydrogels can continuously release antitumor drug doxorubicin and possess injectable and self-healing biological properties (Pandit et al., 2021). A novel amphiphilic chitosan micelle reported to protect 75% of an anticancer drug from hydrolysis is now being used as a promising drug-delivery system (Almeida et al., 2020). The chitosan- and saline-based nanoparticles are used to deliver the pro-oxidant drug piperlongumine to prostate cancer cells due to their prostate cancer cells killing properties (Choi et al., 2019). The antitumor properties of chitosan make it a potential antitumor drug carrier for treating melanoma and sarcoma of skin and soft tissues.

### 3.4 Immunomodulatory Effects

Chitosan and its derivatives can stimulate phagocytes, induce natural killer cells to secrete cytokines, and activate immune-regulatory responses (Moran et al., 2018). The hydrolysate of chitosan can increase the phagocytic activity of macrophages and promote the proliferation of splenocytes and Payer’s patch lymphocytes, thereby exerting unique immunomodulatory properties (Chang et al., 2019). Polymers containing chitosan can promote the polarization of primary bone marrow–derived macrophages to anti-inflammatory activity carrying macrophages (Papadimitriou et al., 2017). Acidified chitosan can provide an immune microenvironment for osteogenic differentiation by promoting crosstalk between the immune cells and stem cells to induce angiogenesis and bone regeneration (Shu et al., 2018). Hydrogels containing chitosan can promote the wound healing capacity of the skin of diabetic rats by downregulating the pro-inflammatory factors like tumor necrosis factor-α and interleukin (IL)-1β (Chen et al., 2021). Chitosan oligosaccharides can promote the phagocytic activity of RAW264.7 cells, produce reactive oxygen species, release pro-inflammatory factors through the NF-КB pathway, and significantly enhance the immunomodulatory effect (Deng et al., 2020). Chitosan can induce and regulate immune cells by altering the microenvironment of the immune system to achieve therapeutic effects by regulating immune function in the skin and soft tissues.

## 4 Drug Carriers Prepared Using Chitosan and its Derivatives

Chitosan has been used to synthesize several drug carriers for drug-delivery systems, such as nanoparticles, films, sponges, hydrogels, and scaffolds. The design of these carriers is based on the biological properties of chitosan and its derivatives. Some of these carriers are currently used in a clinical setting (Supplementary Figure S2).

### 4.1 Nanoparticles

In recent years, nanomaterials have gained increasing attention in the biomedical field (Zhang E. et al., 2019). Chitosan nanoparticles retain the biological properties of chitosan while improving the stability of the loaded drugs and controlling the drug-release rate (Rizeq et al., 2019). There is evidence that chitosan nanoparticles loaded with anticancer drugs could be used to target malignant tumors, thereby prolonging the drug action duration, enhancing the anticancer effect, and reducing toxicity (Assa et al., 2017). Chitosan nanoparticles are safe, biodegradable, and easy to form DNA or protein complexes for use as a potential gene delivery system (Bowman and Leong, 2006). Chitosan-coated silica nanoparticles have been shown to induce a strong immune response *in vivo* and can be used for oral delivery of protein vaccine (Wu et al., 2021). Chitosan nanoparticles retain the biocompatibility and biodegradability of chitosan, which is a valuable property and a promising therapeutic approach in targeted therapy when used in combination with anticancer drugs.

### 4.2 Film

The chitosan-based films possess good permeability, a large surface area, and unique antibacterial properties, thus making them a potential alternative to artificial skin and an important material for wound dressings (Vivcharenko et al., 2020). The surface hydrophobicity, permeability, and sensitivity of gamma ray–irradiated chitosan films can be increased without significant changes in the original chemical structure (Salari et al., 2021). Introducing montmorillonite-copper chloride into chitosan films can increase their tensile strength and elongation at break and also confer higher antibacterial activity against foodborne pathogens, further highlighting their use as a wound dressing to combat infections (Nouri et al., 2018). Additionally, chitosan films containing human epidermal growth factors can protect against enzymatic hydrolysis and endocytosis and significantly accelerate the rate of wound healing in mice (Umar et al., 2021). These antibacterial properties and regenerative effects of chitosan make it a suitable material for wound dressing.

### 4.3 Sponges

The porous structure, biocompatibility, and liquid-absorption properties of the chitosan sponge make it a suitable biomaterial for hemostasis (Zhang K. et al., 2020). Chitosan composite sponges can absorb water in the blood and increase blood viscosity. Moreover, they are non-toxic and biodegradable, hold antibacterial drugs, and promote blood coagulation in wounds (Hu S. et al., 2018). Chitosan composite sponges rich in andrographolide possess a large pore size and expansion rate and can effectively promote wound healing and reduce scar formation when used as a wound care material (Sanad and Abdel-Bar, 2017). Chitosan sponge provides a moist environment, allows gas exchange and blocks out microorganisms, suitable for burn wound dressing to keep away from contamination and dehydration (Jayakumar et al., 2011). Chitosan sponges have been widely used as hemostatic materials due to their porous structure and wound dressings promoting wound healing when loaded with drugs (Matica et al., 2019).

### 4.4 Hydrogels

Hydrogels are hydrophilic polymers with high water content and good biocompatibility. They can be loaded with chitosan and used as wound dressings to keep the wound moist and to continuously absorb exudates (Song et al., 2021). Chitosan hydrogels loaded with metal ions can improve the imbalance in metal ions that cause delayed wound healing. Moreover, they inhibit infections and accelerate healing by regulating the expression of inflammatory factors and macrophages polarization (Xiao et al., 2021). An imbalance in metal ions can also lead to scar growth. Modulating the cation in chitosan hydrogel or adding aloe gel can lead to effective scar inhibition (Zhang N. et al., 2020). Chitosan hydrogels can also be used as hemostatic dressings. Thermal-responsive chitosan/DOPA hydrogels improve the adhesion and show a good hemostatic effect in rats (Shou et al., 2020). Chitosan sponges are often used as a hemostatic material. Hydrogels are commonly used as antibacterial dressings because their hydrophilicity and absorbability can suitably isolate infections from foreign substances and keep the wound moist.

### 4.5 Scaffolds

Tissue engineering is a research hotspot in regenerative medicine. Functional scaffolds composed of natural polymers have been widely used in surgical reconstruction (Rodríguez-Vázquez et al., 2015). Collagen/chitosan scaffolds made using 3D printing technology show remarkable therapeutic effects *in vivo* with complete spinal cord transection, and significantly improve sensory and motor recovery (Sun et al., 2019). Chitosan scaffolds surrounded by microcellulose arranged with twisted polylactic acid can simulate the extracellular matrix of tendons, provide structural support for tendon regeneration, and facilitate tendon-cell attachment and proliferation (Nivedhitha Sundaram et al., 2019). Composite chitosan-gelatin scaffold with a double-tubular structure having large internal pores and nonporous outer layers simulate blood vessels and significantly promote the proliferation of human dermal fibroblasts after being inoculated, and can be used for angiogenesis reconstruction (Badhe et al., 2017). Nano-scaffolds made of chitosan, sulfonated chitosan, polycaprolactone, and phosphoric acid can enhance the activity and adhesion of osteoblasts, making them excellent materials for bone tissue regeneration (Ghaee et al., 2017). Chitosan scaffolds have plastic structure and the ability to promote adhesion and proliferation of tissue cells, improving soft tissue and bone tissue regeneration.

## 5 Applications of Chitosan and its Derivatives to Treat Soft Tissue Diseases

### 5.1 Promotion of Wound Healing

Soft tissue injury refers to laceration and contusion of the skin, subcutaneous tissue, and muscle caused by an external force, bleeding, and local swelling. Wound healing depends on the nature and degree of tissue defects, whereas age, nutritional status, and underlying diseases are systemic factors affecting wound healing (Wilkinson and Hardman, 2020). Promoting wound healing and reducing scar formation are urgent medical problems to be solved for patients with wounds and defects in body function. The antibacterial properties of chitosan and its ability to promote tissue regeneration have increased its usage in wound dressings combined with different materials, which have the overall effect of promoting wound healing (Figure 1B).

Impregnating chitosan hydrogels with silver nanoparticles can significantly improve antibacterial and antioxidant properties and enhance wound healing *in vivo* (Masood et al., 2019). The anti-biofilm formation ability of chitosan-immobilized ficin can inhibit *S. aureus* infections and promote the formation of smoother epithelial tissue (Baidamshina et al., 2020). Vaccinin-chitosan nanoparticles can promote vascular tissue production by upregulating IL-1β and PDGF-BB, thereby highlighting its potential in wound healing (Hou et al., 2020). The curcumin-loaded chitosan membranes can effectively inhibit bacterial pathogens in wounds by increasing the formation of fibrous connective tissue. Additionally, they have an obvious healing effect on wounds resulting from second-degree burns (Abbas et al., 2019). A study reports that macrophage dysfunction can lead to chronic inflammation and inhibit diabetic wound healing (Chen et al., 2021). Chitosan sulfate can improve macrophage function by inducing the polarization of M1 macrophages to M2 macrophages and promoting the production of anti-inflammatory factors, thus effectively promoting diabetic wound healing (Shen et al., 2020). Chitosan has antibacterial, antioxidant, and immunomodulatory effects that can prevent the infection of wounds and promote healing through soft tissue regeneration, making it a natural wound-dressing material.

### 5.2 Anti-Infective Effects in Skin and Soft Tissue

Soft tissue infection is an inflammatory condition caused by pathogenic bacteria that invade the skin and subcutaneous tissue. Elimination of necrotic tissue and pathogenic bacteria is the cornerstone of treatment in such infections (Burnham and Kollef, 2018). The effectiveness of different wound dressings in controlling and treating infection has been clearly demonstrated, highlighting their wide use in clinical practice (Simões et al., 2018). Chitosan is an effective carrier of anti-infective drugs due to its mucous membrane dependence and the ability to prolong drug activity by retarding the biodegradation rate (Rajitha et al., 2016). The inhibitory effects of antibacterial materials based on chitosan and its derivatives on different pathogens are listed in Table 1.

**TABLE 1:** Antibacterial effect of chitosan and its derivatives on different microorganisms.

Polymer	Microbial	Ref
P-COOH-CS-PHMB	*E. coli*	Ng et al. (2020)
Boc-D-Phe-γ 4 -L-Phe-PEA/chitosan	*E. coli*	Malhotra et al. (2020)
CTs@Ag/Sep	*E. coli*	Li et al. (2020)
CS-MoS_2_	*E. coli*	Cao et al. (2019)
Chitosan-sodium phytate nanoparticles	*E. coli*	Yang et al. (2017)
HBCS	*E. coli*	Li et al. (2019)
CS-MCA	*E. coli*	Luo et al. (2019)
CTS/C-Ag	*E. coli*	Hu et al. (2019)
CMCh-Zn	*E. coli*	Wang et al. (2020)
Chitosan-silver nanocomposite	*E. coli*	Raghavendra et al. (2016)
Chitosan/Alkynyl chitosan	*E. coli*	Ding et al. (2013)
PAN-chitosan	*E. coli*	Kim and Lee, (2014)
Chitosan/phosvitin	*E. coli*	Zhou et al. (2014)
CMCh/CuO	*E. coli*	Wahid et al. (2017)
O-CMCS	*E. coli*	He et al. (2016)
CT-TG/SiO_2_	*E. coli*	Mallakpour and Abbasi, (2020)
Chitosan-silver nanoparticles	*E. coli*	Shahid Ul et al. (2019)
Chitosan-g-eugenol/zwitterionic copolymer	*E. coli*	Li et al. (2018)
N-phosphonium chitosan	*E. coli*	Guo et al. (2014)
CS-MnO_2_	*E. coli*	Anwar, (2018)
3,6-O-[N-(2-aminoethyl)-acetamide-yl]-chitosan	*E. coli*	Yan et al. (2016)
Quaternary ammonium chitosan	*E. coli*	Min et al. (2020)
PVA-CS	*E. coli*	Liu et al. (2018)
O-acetyl-chitosan-N-2-hydroxypropyl trimethyl ammonium chloride	*E. coli*	Cai et al. (2015)
Carboxymethyl chitosan/ZnO	*E. coli*	Wahid et al. (2016)
β-chitosan	*E. coli*	Jung et al. (2014)
Carboxymethyl chitosan	*E. coli*	Olanipekun et al. (2021)
Chi-Ag NPs	*E. coli*	Senthilkumar et al. (2019)
Carboxymethyl chitosan-zinc supramolecular hydrogels	*E. coli*	Wahid et al. (2018)
Chitosan-g-poly acrylonitrile/silver nanocomposite	*E. coli*	Hebeish et al. (2014)
Quaternized carboxymethyl chitosan	*E. coli*	Yin et al. (2018)
CH-CL	*S. aureus*	Wang et al. (2021)
Boc-D-Phe-γ 4 -L-Phe-PEA/chitosan	*S. aureus*	Malhotra et al. (2020)
CTs@Ag/Sep	*S. aureus*	Li et al. (2020)
CS-MoS_2_	*S. aureus*	Cao et al. (2019)
HBCS	*S. aureus*	Li et al. (2019)
CMCh-Zn	*S. aureus*	Wang et al. (2020)
Chitosan-silver nanocomposite films	*S. aureus*	Raghavendra et al. (2016)
N-quaternary chitosan	*S. aureus*	Ghazaie et al. (2019)
Chitosan/Alkynyl chitosan	*S. aureus*	Ding et al. (2013)
PAN-chitosan	*S. aureus*	Kim and Lee, (2014)
Chitosan/phosvitin	*S. aureus*	Zhou et al. (2014)
CMCh/CuO	*S. aureus*	Wahid et al. (2017)
O-CMCS	*S. aureus*	He et al. (2016)
CT-TG/SiO_2_	*S. aureus*	Mallakpour and Abbasi, (2020)
Chitosan-silver nanoparticles	*S. aureus*	Shahid Ul et al. (2019)
Chitosan-g-eugenol/zwitterionic copolymer	*S. aureus*	Li et al. (2018)
N-phosphonium chitosan	*S. aureus*	Guo et al. (2014)
CS-MnO_2_	*S. aureus*	Anwar, (2018)
CuS/PVACS	*S. aureus*	Wang and Fakhri, (2020)
3,6-O-[N-(2-aminoethyl)-acetamide-yl]-chitosan	*S. aureus*	Yan et al. (2016)
N, N, N-Trimethyl Chitosan	*S. aureus*	Sahariah et al. (2019)
Quaternary ammonium chitosan	*S. aureus*	Min et al. (2020)
PVA-CS	*S. aureus*	Liu et al. (2018)
Surface-quaternized chitosan particles	*S. aureus*	Wiarachai et al. (2012)
O-acetyl-chitosan-N-2-hydroxypropyl trimethyl ammonium chloride	*S. aureus*	Cai et al. (2015)
Carboxymethyl chitosan/ZnO	*S. aureus*	Wahid et al. (2016)
Chitosan-silver nanocomposites	*S. aureus*	Potara et al. (2011)
NAM-CMCS-ZnO	*S. aureus*	Rao et al. (2020)
MDAACS	*S. aureus*	Jou, (2011)
Chitosan-gold nanocomposites	*S. aureus*	Regiel-Futyra et al. (2015)
Carboxymethyl chitosan-zinc supramolecular hydrogels	*S. aureus*	Wahid et al. (2018)
Ferulic acid-grafted chitosan	*S. aureus*	Dasagrandhi et al. (2018)
Chitosan-g-poly acrylonitrile/silver nanocomposite	*S. aureus*	Hebeish et al. (2014)
Quaternized carboxymethyl chitosan	*S. aureus*	Yin et al. (2018)
Carboxymethyl chitosan	*Pseudomonas aeruginosa*	Olanipekun et al. (2021)
Boc-D-Phe-γ 4 -L-Phe-PEA/chitosan	*Pseudomonas aeruginosa*	Malhotra et al. (2020)
Chitosan-gold nanocomposites	*Pseudomonas aeruginosa*	Regiel-Futyra et al. (2015)
Ferulic acid-grafted chitosan	*Pseudomonas aeruginosa*	Dasagrandhi et al. (2018)
β-chitosan	*Listeria innocua*	Jung et al. (2014)
Ferulic acid-grafted chitosan	*Listeria innocua*	Dasagrandhi et al. (2018)
Carboxymethyl chitosan	*Klebsiella Pneumoniae*	Olanipekun et al. (2021)
MDAACS	*Klebsiella Pneumoniae*	Jou, (2011)
CTs@Ag/Sep	*Aspergillus niger*	Li et al. (2020)
Chitosan-glutaraldehyde	*Burkholderia cepacia*	Li et al. (2013)
PAN-chitosan	*Micrococcus luteus*	Kim and Lee, (2014)
CuS/PVACS	*Streptococcus pneumonia*	Wang and Fakhri, (2020)
Quaternary ammonium chitosan	*Botrytis cinerea*	Min et al. (2020)
CNPs	*N. gonorrhoeae*	Alqahtani et al. (2020)

### 5.3 Promotion of Soft Tissue Regeneration

#### 5.3.1 Skin Regeneration

Skin injuries or necrosis caused by crush, burn, or cut injuries are medical problems warranting urgent care. Common treatment methods include autogenous skin transplantation and free or pedicled skin-flap transplantation, which can cause problems, such as graft tissue necrosis, scar contracture, and poor cosmetic appearance (Przekora, 2020; Li et al., 2021). The tissue-repair function of chitosan provides a novel solution for skin reconstruction (Wei et al., 2022). Hydrogels synthesized from chitosan and cellulose can accelerate epithelial tissue formation on wounds and mimic skin structure, induce skin regeneration, and can be loaded with antibacterial agents to prevent wound infections (Alven and Aderibigbe, 2020). Lithium chloride–loaded chitosan hydrogels can significantly reduce wound inflammation, promote angiogenesis, and accelerate epithelial regeneration, thereby showing a potential dressing for skin regeneration (Yuan et al., 2020). Chitosan wound dressings containing exosomes derived from overexpressed miRNA-126 synovial mesenchymal stem cells can promote epithelium formation, angiogenesis, and collagen maturation in diabetic rats (Tao et al., 2017). Chitosan can promote skin regeneration by promoting angiogenesis and epithelium formation.

#### 5.3.2 Tendon Regeneration

Tendons are one of the major components responsible for maintaining the movement of various joints in the body. Tendon rupture due to trauma can lead to irreversible impaired movement. The tendon structure simulated by poly (l-lactic acid) nanofibers can promote the regeneration of the broken flexor tendons and alginate gel, a novel natural biological scaffold suitable for tendon repair in the outer layer, and can prevent tendon adhesion (Deepthi et al., 2016). Asymmetric chitosan scaffolds have been developed to encapsulate rat tendon stem/progenitor cells and promote tendon regeneration (Chen et al., 2018). The polycaprolactone/chitosan nanofiber biocomposite prepared using the electrostatic spinning process can promote the adhesion and proliferation of human osteoblasts and be used for tendon and ligament regeneration (Wu et al., 2018). Biomaterials based on chitosan and its derivatives can promote tendon healing and prevent adhesion around tendons, which is beneficial for treating patients with tendon rupture.

#### 5.3.3 Nerve Regeneration

Peripheral nerves are the nerves outside the brain and spinal cord. Damage to these nerves can lead to motor and sensory impairments. The biological materials with chitosan as the primary polymer are effective in nerve-injury repair. The related mechanisms are shown in Figure 1C. Chitosan nanofiber hydrogels prepared by electrospinning and mechanical stretching can stimulate brain-derived neurotrophic factor and vascular endothelial growth factor, promote Schwann cell proliferation, and secrete neurotrophic silver to repair sciatic nerve defects in the sciatic nerve–defect model of mice (Rao F. et al., 2020). Additionally, sciatic nerve defects in rats were repaired using a nerve catheter containing chitosan reinforced with chitosan membrane in the longitudinal direction, and the result was anastomosed with autologous nerve transplantation (Meyer et al., 2016). Heparin/chitosan scaffolds loaded with nerve growth factors through electrostatic interaction can significantly promote the morphological development of Schwann cells and exhibit good stability (Li et al., 2017). The effective proliferation of Schwann cells accelerates the rate of nerve regeneration. Chitosan derivatives can affect nerve regeneration through immunomodulatory effects. As a degradation product of chitosan, chitosan oligosaccharides can promote nerve regeneration by regulating the microenvironment of macrophages infiltrating around injured sciatic nerves (Zhao et al., 2017). Compared with traditional surgical repair techniques, chitosan and its derivatives are more coherent for soft tissues regeneration, with less damage, easier acquisition, and more satisfying outcomes.

### 5.4 Promotion of Coagulation

Bleeding due to trauma is a serious symptom that needs immediate attention during surgical emergencies. Chitosan can promote coagulation by enhancing red blood cell agglutination and platelet adhesion and is a potential hemostatic material (Figure 1D) (Hu Z. et al., 2018). Composite sponges containing alginate/carboxymethyl chitosan/kangfuxin are biodegradable materials that accelerate blood clotting and promote wound closure (He et al., 2021). Carboxymethyl chitosan sponges grafted with marine collagen peptides can promote coagulation both *in vivo* and *in vitro* through the synergistic effect of the collagen peptide and carboxymethyl chitosan (Cheng et al., 2020). Chitosan/diatom-biosilica aerogels are associated with large surface areas and excellent water absorption capabilities and hence, show the shortest clotting time and the lowest amount of blood loss in a hemorrhage model of rats (Li J. et al., 2020). Chitosan/cellulose composite sponges with LiOH/KOH/urea solvent in the shell show better clotting ability, antibacterial effect, and good absorbability than traditional gauze and gelatin sponges (Fan et al., 2020). Different chitosan materials exhibit varying absorbability and coagulation-promoting effects and serve as convenient and effective hemostatic materials to arrest acute bleeding of the skin and soft tissues.

### 5.5 Targeted Therapy for Soft Tissue Malignancy

Soft tissue malignancy or sarcomas are tumors that originated from the mesenchymal tissue and mainly occur in the muscles, ligaments, periosteum, fat, and other sites. The efficacy of chitosan in drug-delivery systems for the targeted therapy of malignant tumors in sarcoma has been well documented (Tan et al., 2010). Methylglyoxal-conjugated chitosan nanoparticles can enhance the anticancer effect of methylglyoxal alone in tumor-bearing mice and protect it from enzymatic degradation *in vivo* by upregulating cytokines and surface receptors of macrophages (Chakrabarti et al., 2014; Pal et al., 2015). Thus, the immunomodulatory effects of macrophages should be activated to achieve the antitumor effect. Low-molecular-weight chitosan obtained through enzymolysis can increase the natural killing activity of tumor-bearing intestinal intraepithelial lymphocytes in mice and inhibit tumor growth by activating their intestinal immune function (Maeda and Kimura, 2004), suggesting that chitosan can achieve antitumor effects by regulating the immune system. Additionally, chitosan can reduce gastrointestinal tract injury caused by adriamycin in sarcoma-180–bearing mice without affecting the tumor-inhibition effect (Kimura et al., 2001). Chitosan can be used to prevent weight loss and spleen weight loss caused by cisplatin in tumor-bearing mice without reducing the antitumor activity of the drug (Kimura et al., 2000). Therefore, chitosan can be considered to alleviate the toxic and side effects of chemotherapy in individuals with sarcoma. Chitosan can increase the anticancer effect of drugs, reduce damage to the body, and achieve antitumor effects through immune regulation when used as a targeted drug carrier. These factors highlight its usage as a curative material in treating soft tissue tumors.

## 6 Discussion

Chitosan and its derivatives exhibit good biocompatibility. They are biodegradable, nontoxic, and also exert antibacterial, antioxidant, antitumor, and immunomodulatory effects. Chitosan can be used to synthesize different types of drug carriers based on the intended use, as it plays a significant role in soft tissue diseases treatment (Supplementary Table S1) (Wang W. et al., 2020). Chitosan nanoparticles can improve drug stability while retaining the biological properties of chitosan, thereby rendering them suitable as carriers of targeted drugs (Aibani et al., 2021). Chitosan nanoparticles are associated with fewer drug-loading and biological distribution limitations compared with lipid-based nanoparticles. Moreover, chitosan nanoparticles are nontoxic and not radioactive as inorganic nanoparticles (Dadfar et al., 2019; Alphandéry, 2020; Plucinski et al., 2021). Chitosan films can be made into antibacterial dressings to enhance the antibacterial effect of chitosan (Rashki et al., 2021). Skin irritation or local side effects are rare due to the biodegradability and biocompatibility of chitosan. Thus, the incidence of contact dermatitis is lesser with the use of chitosan than with the use of traditional antibacterial agents (Homaeigohar and Boccaccini, 2020; Zheng et al., 2020). Chitosan sponges possess good absorbability and a porous structure and are not associated with immunogenicity and virality compared with other thrombin- and fibrin-based products (Yu and Zhong, 2021). Chitosan sponges are degraded *in vivo* after exerting their hemostatic role; these sponges are less toxic and exhibit fewer side effects than mineral hemostatic materials (Hickman et al., 2018). Chitosan hydrogels have a high-water content, which can keep wounds moist and prevent secondary damage caused by traditional gauze while changing dressings (Thapa et al., 2020). The drug-loaded chitosan hydrogels can slowly release drugs and prevent tissue damage caused by the burst effect due to sudden drug release (Teixeira et al., 2021). The ductility and absorbability of chitosan hydrogels render them suitable for application to limb injuries and avoid sliding of the dressing and wound exposure caused by joint movement (Zhang A. et al., 2020). Chitosan scaffolds are important components in bone tissue engineering. They can be used to repair bone defects and carry mesenchymal stem cells for nerve and tendon regeneration, which is a major breakthrough in regenerative medicine (Cofano et al., 2019; Zhang L. et al., 2019; Vijayavenkataraman, 2020; Russo et al., 2022). Compared with other drug carriers, chitosan and its derivatives could be a potential approach for preventing and treating of skin and soft tissue diseases.

Bacterial resistance limits the systemic effects of antibiotics and is one of the major factors delaying the healing of chronic infections of the skin and soft tissues (Theuretzbacher et al., 2020). Chitosan can directly interact with bacteria at the site of infection to exert antibacterial effects and eradicate the infection at the site (Jyoti et al., 2020). Chitosan can regulate the immune microenvironment of the body, activate immune cells, and exert anti-infective effects by enhancing immunity (Moran et al., 2018). Compared with silver nanoparticles, chitosan exhibits better antibacterial properties while promoting tissue regeneration (Tang and Zheng, 2018), making it more suitable as an antibacterial agent to treat skin and soft tissue infections. For bleeding caused by skin and soft tissue trauma, compression or tourniquet is often used to stop bleeding. However, this method has limited hemostatic effect and is easy to form thrombus and hematoma (Weiskopf, 2009). Chitosan and its derivatives can stop bleeding by inducing erythrocyte agglutination and platelet adhesion, thereby accelerating blood coagulation and promoting wound healing (He et al., 2021). However, there is little evidence on whether chitosan hemostatic material can induce thrombosis. At present, soft tissue sarcomas treatment relies on surgery. For patients who cannot suffer from surgery, radiotherapy and chemotherapy become the first choices (Hoefkens et al., 2016). Chitosan and its derivatives can carry anti-tumor drugs to achieve a targeted treatment of soft tissue sarcoma, which can increase the anti-tumor efficiency of drugs and reduce the toxicity and side effects (Kimura et al., 2000). The role of chitosan in bone tissue engineering has been widely studied, but there is little evidence of the skin and soft tissue regeneration (Ghaee et al., 2017). Therefore, studies should pay more attention to the chitosan regeneration on the skin and soft tissue, especially peripheral nerves, as nerves take a long time to regenerate and are more prone to secondary rupture.

In conclusion, as a natural polymer, chitosan and its derivatives have been isolated from a wide range of sources. The advantages include ease of preparation and good biological characteristics, which are useful attributes in the prevention and treatment of soft tissue diseases.
