# Corrigendum: Applications of chitosan and its derivatives in skin and soft tissue diseases

**DOI:** 10.3389/fbioe.2022.1082945

**Published:** 2022-11-25

**Authors:** Yidan Xia, Dongxu Wang, Da Liu, Jiayang Su, Ye Jin, Duo Wang, Beibei Han, Ziping Jiang, Bin Liu

**Affiliations:** ^1^ Department of Hand and Foot Surgery, The First Hospital of Jilin University, Changchun, China; ^2^ Laboratory Animal Center, College of Animal Science, Jilin University, Changchun, China; ^3^ Department of Pharmacy, Changchun University of Chinese Medicine, Changchun, China

**Keywords:** chitosan, soft tissue disease, biological property, drug-delivery carrier, regenerative medicine

In the published article, there was an error in Table 1 as published. The references in Table 1 were incorrectly presented. The corrected Table 1 and its caption appear below.

**TABLE 1:** Antibacterial effect of chitosan and its derivatives on different microorganisms.

Polymer	Microbial	Ref
P-COOH-CS-PHMB	*E. coli*	Ng et al. (2020)
Boc-D-Phe-γ 4 -L-Phe-PEA/chitosan	*E. coli*	Malhotra et al. (2020)
CTs@Ag/Sep	*E. coli*	Li et al. (2020)
CS-MoS_2_	*E. coli*	Cao et al. (2019)
Chitosan-sodium phytate nanoparticles	*E. coli*	Yang et al. (2017)
HBCS	*E. coli*	Li et al. (2019)
CS-MCA	*E. coli*	Luo et al. (2019)
CTS/C-Ag	*E. coli*	Hu et al. (2019)
CMCh-Zn	*E. coli*	Wang et al. (2020)
Chitosan-silver nanocomposite	*E. coli*	Raghavendra et al. (2016)
Chitosan/Alkynyl chitosan	*E. coli*	Ding et al. (2013)
PAN-chitosan	*E. coli*	Kim and Lee, (2014)
Chitosan/phosvitin	*E. coli*	Zhou et al. (2014)
CMCh/CuO	*E. coli*	Wahid et al. (2017)
O-CMCS	*E. coli*	He et al. (2016)
CT-TG/SiO_2_	*E. coli*	Mallakpour and Abbasi, (2020)
Chitosan-silver nanoparticles	*E. coli*	Shahid Ul et al. (2019)
Chitosan-g-eugenol/zwitterionic copolymer	*E. coli*	Li et al. (2018)
N-phosphonium chitosan	*E. coli*	Guo et al. (2014)
CS-MnO_2_	*E. coli*	Anwar, (2018)
3,6-O-[N-(2-aminoethyl)-acetamide-yl]-chitosan	*E. coli*	Yan et al. (2016)
Quaternary ammonium chitosan	*E. coli*	Min et al. (2020)
PVA-CS	*E. coli*	Liu et al. (2018)
O-acetyl-chitosan-N-2-hydroxypropyl trimethyl ammonium chloride	*E. coli*	Cai et al. (2015)
Carboxymethyl chitosan/ZnO	*E. coli*	Wahid et al. (2016)
β-chitosan	*E. coli*	Jung et al. (2014)
Carboxymethyl chitosan	*E. coli*	Olanipekun et al. (2021)
Chi-Ag NPs	*E. coli*	Senthilkumar et al. (2019)
Carboxymethyl chitosan-zinc supramolecular hydrogels	*E. coli*	Wahid et al. (2018)
Chitosan-g-poly acrylonitrile/silver nanocomposite	*E. coli*	Hebeish et al. (2014)
Quaternized carboxymethyl chitosan	*E. coli*	Yin et al. (2018)
CH-CL	*S. aureus*	Wang et al. (2021)
Boc-D-Phe-γ 4 -L-Phe-PEA/chitosan	*S. aureus*	Malhotra et al. (2020)
CTs@Ag/Sep	*S. aureus*	Li et al. (2020)
CS-MoS_2_	*S. aureus*	Cao et al. (2019)
HBCS	*S. aureus*	Li et al. (2019)
CMCh-Zn	*S. aureus*	Wang et al. (2020)
Chitosan-silver nanocomposite films	*S. aureus*	Raghavendra et al. (2016)
N-quaternary chitosan	*S. aureus*	Ghazaie et al. (2019)
Chitosan/Alkynyl chitosan	*S. aureus*	Ding et al. (2013)
PAN-chitosan	*S. aureus*	Kim and Lee, (2014)
Chitosan/phosvitin	*S. aureus*	Zhou et al. (2014)
CMCh/CuO	*S. aureus*	Wahid et al. (2017)
O-CMCS	*S. aureus*	He et al. (2016)
CT-TG/SiO_2_	*S. aureus*	Mallakpour and Abbasi, (2020)
Chitosan-silver nanoparticles	*S. aureus*	Shahid Ul et al. (2019)
Chitosan-g-eugenol/zwitterionic copolymer	*S. aureus*	Li et al. (2018)
N-phosphonium chitosan	*S. aureus*	Guo et al. (2014)
CS-MnO_2_	*S. aureus*	Anwar, (2018)
CuS/PVACS	*S. aureus*	Wang and Fakhri, (2020)
3,6-O-[N-(2-aminoethyl)-acetamide-yl]-chitosan	*S. aureus*	Yan et al. (2016)
N, N, N-Trimethyl Chitosan	*S. aureus*	Sahariah et al. (2019)
Quaternary ammonium chitosan	*S. aureus*	Min et al. (2020)
PVA-CS	*S. aureus*	Liu et al. (2018)
Surface-quaternized chitosan particles	*S. aureus*	Wiarachai et al. (2012)
O-acetyl-chitosan-N-2-hydroxypropyl trimethyl ammonium chloride	*S. aureus*	Cai et al. (2015)
Carboxymethyl chitosan/ZnO	*S. aureus*	Wahid et al. (2016)
Chitosan-silver nanocomposites	*S. aureus*	Potara et al. (2011)
NAM-CMCS-ZnO	*S. aureus*	Rao et al. (2020)
MDAACS	*S. aureus*	Jou, (2011)
Chitosan-gold nanocomposites	*S. aureus*	Regiel-Futyra et al. (2015)
Carboxymethyl chitosan-zinc supramolecular hydrogels	*S. aureus*	Wahid et al. (2018)
Ferulic acid-grafted chitosan	*S. aureus*	Dasagrandhi et al. (2018)
Chitosan-g-poly acrylonitrile/silver nanocomposite	*S. aureus*	Hebeish et al. (2014)
Quaternized carboxymethyl chitosan	*S. aureus*	Yin et al. (2018)
Carboxymethyl chitosan	*Pseudomonas aeruginosa*	Olanipekun et al. (2021)
Boc-D-Phe-γ 4 -L-Phe-PEA/chitosan	*Pseudomonas aeruginosa*	Malhotra et al. (2020)
Chitosan-gold nanocomposites	*Pseudomonas aeruginosa*	Regiel-Futyra et al. (2015)
Ferulic acid-grafted chitosan	*Pseudomonas aeruginosa*	Dasagrandhi et al. (2018)
β-chitosan	*Listeria innocua*	Jung et al. (2014)
Ferulic acid-grafted chitosan	*Listeria innocua*	Dasagrandhi et al. (2018)
Carboxymethyl chitosan	*Klebsiella Pneumoniae*	Olanipekun et al. (2021)
MDAACS	*Klebsiella Pneumoniae*	Jou, (2011)
CTs@Ag/Sep	*Aspergillus niger*	Li et al. (2020)
Chitosan-glutaraldehyde	*Burkholderia cepacia*	Li et al. (2013)
PAN-chitosan	*Micrococcus luteus*	Kim and Lee, (2014)
CuS/PVACS	*Streptococcus pneumonia*	Wang and Fakhri, (2020)
Quaternary ammonium chitosan	*Botrytis cinerea*	Min et al. (2020)
CNPs	*N. gonorrhoeae*	Alqahtani et al. (2020)

The authors apologize for this error and state that this does not change the scientific conclusions of the article in any way. The original article has been updated.
