# Supplementary material for: Applications of Chitosan and its Derivatives in Skin and Soft Tissue Diseases
Source: Front Bioeng Biotechnol. 2022 May 2;10:894667. doi: 10.3389/fbioe.2022.894667 (PMC9108203; doi:10.3389/fbioe.2022.894667)
Supplement: Supplementary file 1 [file Table1.DOCX]

Table.S1

Therapeutic effects of chitosan and its derivatives in soft tissue diseases

| **Functions** | **Chitosan** | **Ref** |
| --- | --- | --- |
| Promotion of wound healing | Impregnating chitosan hydrogels with silver nanoparticles | (1) |
|  | Chitosan-immobilized ficin | (2) |
|  | Vaccinin-chitosan nanoparticles | (3) |
|  | Curcumin-loaded chitosan membranes | (4) |
|  | Chitosan sulfate | (5) |
| Skin regeneration | Hydrogels synthesized from chitosan and cellulose | (6) |
|  | Lithium chloride–loaded chitosan hydrogels | (7) |
|  | Chitosan exosomes derived from overexpressed miRNA-126 synovial mesenchymal stem cells | (8) |
| Tendon regeneration | Poly (l-lactic acid) nanofibers | (9) |
|  | Asymmetric chitosan scaffolds | (10) |
|  | Polycaprolactone/chitosan nanofiber biocomposite | (11) |
| Nerve regeneration | Chitosan nanofiber hydrogels | (12) |
|  | Chitosan nerve catheter | (13) |
|  | Heparin/chitosan scaffolds | (14) |
| Promotion of coagulation | Composite sponges containing alginate/carboxymethyl chitosan/ kangfuxin | (15) |
|  | Carboxymethyl chitosan sponges grafted with marine collagen peptides | (16) |
|  | Chitosan/diatom-biosilica aerogels | (17) |
|  | Chitosan/cellulose composite sponges with LiOH/KOH/urea solvent | (18) |
| Targeted therapy for malignancy | Methylglyoxal-conjugated chitosan nanoparticles | (19, 20) |
|  | Chitosan | (21, 22) |
|  | Low-molecular-weight chitosan | (23) |

References

1. Masood N, Ahmed R, Tariq M, Ahmed Z, Masoud MS, Ali I, et al. Silver nanoparticle impregnated chitosan-PEG hydrogel enhances wound healing in diabetes induced rabbits. Int J Pharm. 2019;559:23-36.

2. Baidamshina DR, Koroleva VA, Trizna EY, Pankova SM, Agafonova MN, Chirkova MN, et al. Anti-biofilm and wound-healing activity of chitosan-immobilized Ficin. Int J Biol Macromol. 2020;164:4205-17.

3. Hou B, Qi M, Sun J, Ai M, Ma X, Cai W, et al. Preparation, characterization and wound healing effect of vaccarin-chitosan nanoparticles. Int J Biol Macromol. 2020;165(Pt B):3169-79.

4. Abbas M, Hussain T, Arshad M, Ansari AR, Irshad A, Nisar J, et al. Wound healing potential of curcumin cross-linked chitosan/polyvinyl alcohol. Int J Biol Macromol. 2019;140:871-6.

5. Shen T, Dai K, Yu Y, Wang J, Liu C. Sulfated chitosan rescues dysfunctional macrophages and accelerates wound healing in diabetic mice. Acta Biomater. 2020;117:192-203.

6. Alven S, Aderibigbe BA. Chitosan and Cellulose-Based Hydrogels for Wound Management. Int J Mol Sci. 2020;21(24).

7. Yuan J, Hou Q, Chen D, Zhong L, Dai X, Zhu Z, et al. Chitosan/LiCl composite scaffolds promote skin regeneration in full-thickness loss. Sci China Life Sci. 2020;63(4):552-62.

8. Tao SC, Guo SC, Li M, Ke QF, Guo YP, Zhang CQ. Chitosan Wound Dressings Incorporating Exosomes Derived from MicroRNA-126-Overexpressing Synovium Mesenchymal Stem Cells Provide Sustained Release of Exosomes and Heal Full-Thickness Skin Defects in a Diabetic Rat Model. Stem Cells Transl Med. 2017;6(3):736-47.

9. Deepthi S, Nivedhitha Sundaram M, Deepti Kadavan J, Jayakumar R. Layered chitosan-collagen hydrogel/aligned PLLA nanofiber construct for flexor tendon regeneration. Carbohydr Polym. 2016;153:492-500.

10. Chen E, Yang L, Ye C, Zhang W, Ran J, Xue D, et al. An asymmetric chitosan scaffold for tendon tissue engineering: In vitro and in vivo evaluation with rat tendon stem/progenitor cells. Acta Biomater. 2018;73:377-87.

11. Wu G, Deng X, Song J, Chen F. Enhanced biological properties of biomimetic apatite fabricated polycaprolactone/chitosan nanofibrous bio-composite for tendon and ligament regeneration. J Photochem Photobiol B. 2018;178:27-32.

12. Rao F, Wang Y, Zhang D, Lu C, Cao Z, Sui J, et al. Aligned chitosan nanofiber hydrogel grafted with peptides mimicking bioactive brain-derived neurotrophic factor and vascular endothelial growth factor repair long-distance sciatic nerve defects in rats. Theranostics. 2020;10(4):1590-603.

13. Meyer C, Stenberg L, Gonzalez-Perez F, Wrobel S, Ronchi G, Udina E, et al. Chitosan-film enhanced chitosan nerve guides for long-distance regeneration of peripheral nerves. Biomaterials. 2016;76:33-51.

14. Li G, Xiao Q, Zhang L, Zhao Y, Yang Y. Nerve growth factor loaded heparin/chitosan scaffolds for accelerating peripheral nerve regeneration. Carbohydr Polym. 2017;171:39-49.

15. He Y, Zhao W, Dong Z, Ji Y, Li M, Hao Y, et al. A biodegradable antibacterial alginate/carboxymethyl chitosan/Kangfuxin sponges for promoting blood coagulation and full-thickness wound healing. Int J Biol Macromol. 2021;167:182-92.

16. Cheng Y, Lu S, Hu Z, Zhang B, Li S, Hong P. Marine collagen peptide grafted carboxymethyl chitosan: Optimization preparation and coagulation evaluation. Int J Biol Macromol. 2020;164:3953-64.

17. Li J, Sun X, Zhang K, Yang G, Mu Y, Su C, et al. Chitosan/Diatom-Biosilica Aerogel with Controlled Porous Structure for Rapid Hemostasis. Adv Healthc Mater. 2020;9(21):e2000951.

18. Fan X, Li Y, Li N, Wan G, Ali MA, Tang K. Rapid hemostatic chitosan/cellulose composite sponge by alkali/urea method for massive haemorrhage. Int J Biol Macromol. 2020;164:2769-78.

19. Chakrabarti A, Talukdar D, Pal A, Ray M. Immunomodulation of macrophages by methylglyoxal conjugated with chitosan nanoparticles against Sarcoma-180 tumor in mice. Cell Immunol. 2014;287(1):27-35.

20. Pal A, Talukdar D, Roy A, Ray S, Mallick A, Mandal C, et al. Nanofabrication of methylglyoxal with chitosan biopolymer: a potential tool for enhancement of its anticancer effect. Int J Nanomedicine. 2015;10:3499-518.

21. Kimura Y, Sawai N, Okuda H. Antitumour activity and adverse reactions of combined treatment with chitosan and doxorubicin in tumour-bearing mice. J Pharm Pharmacol. 2001;53(10):1373-8.

22. Kimura Y, Onoyama M, Sera T, Okuda H. Antitumour activity and side effects of combined treatment with chitosan and cisplatin in sarcoma 180-bearing mice. J Pharm Pharmacol. 2000;52(7):883-90.

23. Maeda Y, Kimura Y. Antitumor effects of various low-molecular-weight chitosans are due to increased natural killer activity of intestinal intraepithelial lymphocytes in sarcoma 180-bearing mice. J Nutr. 2004;134(4):945-50.
