# Supplementary figures and images for: Applications of Chitosan and its Derivatives in Skin and Soft Tissue Diseases
Source: Front Bioeng Biotechnol. 2022 May 2;10:894667. doi: 10.3389/fbioe.2022.894667 (PMC9108203; doi:10.3389/fbioe.2022.894667)

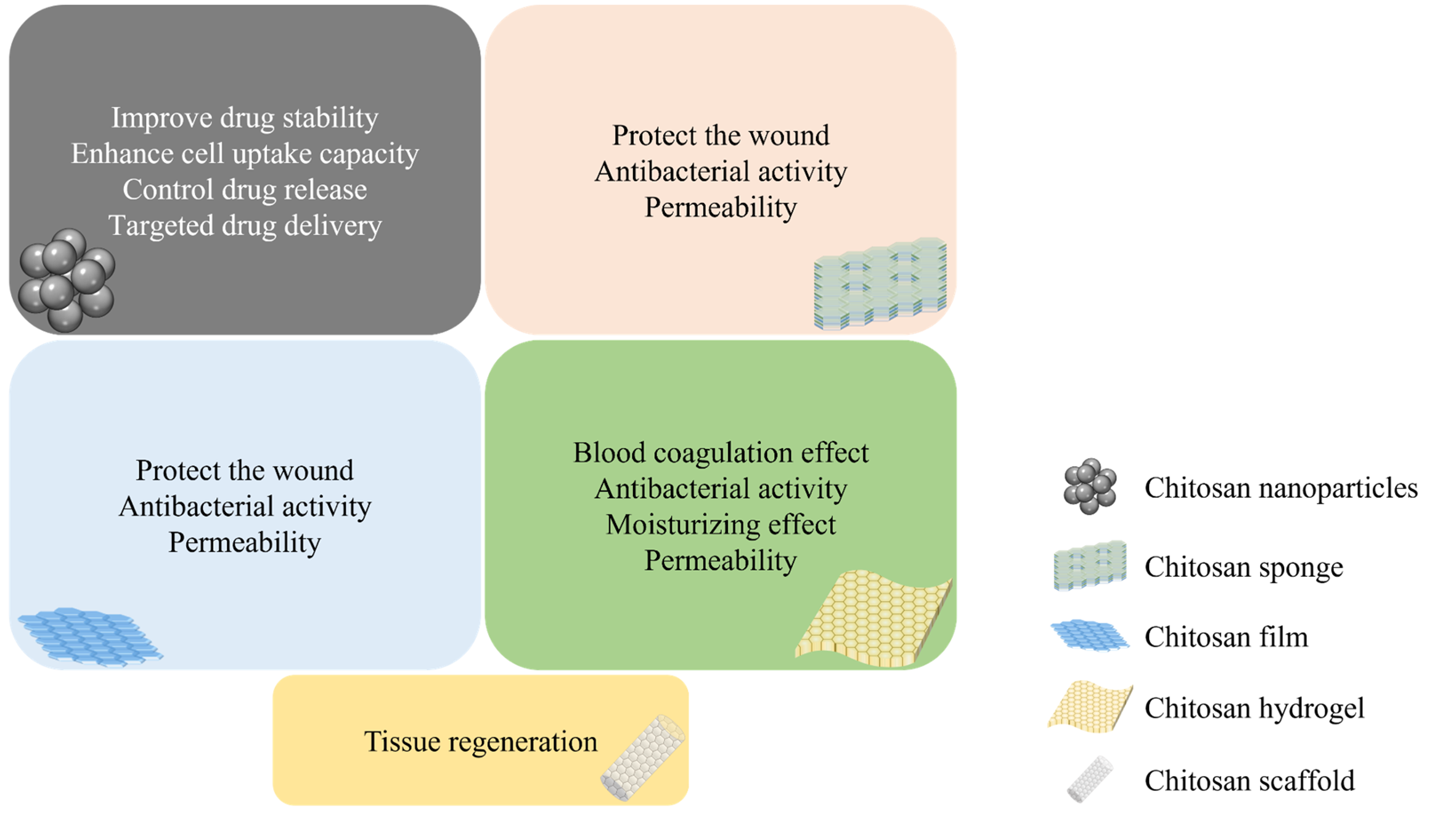

Supplement: Supplementary file 2 [file Image2.TIF]

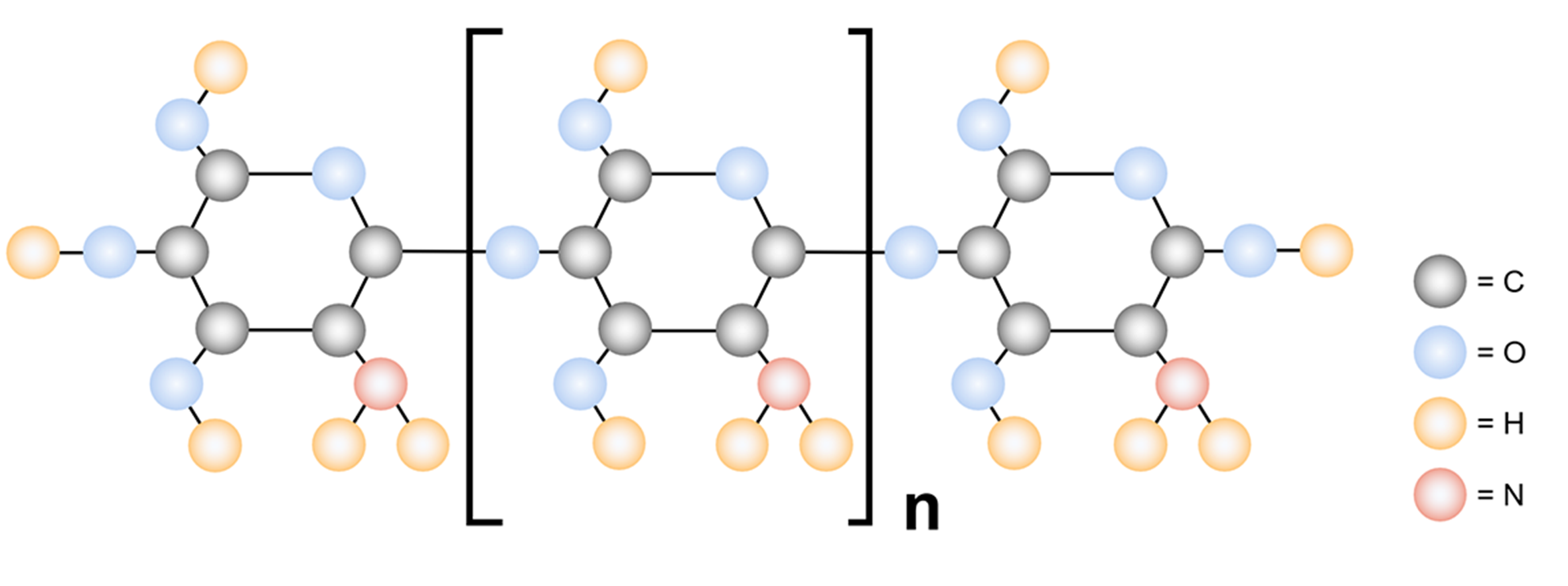

Supplement: Supplementary file 3 [file Image1.TIF]
